# The Psychometric Properties of the Chinese eHealth Literacy Scale (C-eHEALS) in a Chinese Rural Population: Cross-Sectional Validation Study

**DOI:** 10.2196/15720

**Published:** 2019-10-22

**Authors:** Zhihao Ma, Mei Wu

**Affiliations:** 1 Computational Communication Collaboratory School of Journalism and Communication Nanjing University Nanjing China; 2 Department of Communication Faculty of Social Sciences University of Macau Macau China

**Keywords:** eHealth literacy, eHEALS, psychometrics, classical test theory, item response theory

## Abstract

**Background:**

The eHealth Literacy Scale (eHEALS) is the most widely used instrument in health studies to measure individual’s electronic health literacy. Nonetheless, despite the rapid development of the online medical industry and increased rural-urban disparities in China, very few studies have examined the characteristics of the eHEALS among Chinese rural people by using modern psychometric methods. This study evaluated the psychometric properties of eHEALS in a Chinese rural population by using both the classical test theory and item response theory methods.

**Objective:**

This study aimed to develop a simplified Chinese version of the eHEALS (C-eHEALS) and evaluate its psychometric properties in a rural population.

**Methods:**

A cross-sectional survey was conducted with 543 rural internet users in West China. The internal reliability was assessed using the Cronbach alpha coefficient. A one-factor structure of the C-eHEALS was obtained via principal component analysis, and fit indices for this structure were calculated using confirmatory factory analysis. Subsequently, the item discrimination, difficulty, and test information were estimated via the graded response model. Additionally, the criterion validity was confirmed through hypothesis testing.

**Results:**

The C-eHEALS has good reliability. Both principal component analysis and confirmatory factory analysis showed that the scale has a one-factor structure. The graded response model revealed that all items of the C-eHEALS have response options that allow for differentiation between latent trait levels and the capture of substantial information regarding participants’ ability.

**Conclusions:**

The findings indicate the high reliability and validity of the C-eHEALS and thus recommend its use for measuring eHealth literacy among the Chinese rural population.

## Introduction

China has the world’s largest population of internet users, who also frequently access medical resources over the internet [1]. In fact, 26.6% of Chinese internet users use the internet to access online medical service [1]. Internet users adopt online medical platforms and related applications for evaluation of doctors [2], medical inquires [3], and health management [4]. The Chinese government and health practitioners have recognized challenges and opportunities, given the increasing adoption and diffusion of information communication technologies (ICTs) in the health care system [5]. For example, limited health literacy has been shown to pose problems for Chinese residents who access online health resources [6] and has even led to avoidable medical tragedies [7]. Promoting public health literacy in today’s information age is an urgent need in China. Therefore, in 2019, the National Health Commission of the People’s Republic of China proposed the *Health China Campaign (2019-2030)*, which includes the ambitious goal of enhancing residents’ health literacy to decrease the disease burden and improve national well-being [8].

Given the low-cost and accessibility of ICTs, it is not surprising that policymakers, practitioners, and researchers have focused on electronic health (eHealth) and its applications [9]. Thus, for potential and current individual eHealth users, their eHealth literacy should be considered. eHealth literacy refers to “the ability to seek, find, understand, and appraise health information from electronic sources and apply the knowledge gained to addressing or solving a health problem [10].” Norman and Skinner [10] used the Lily model to describe eHealth literacy in six dimensions: computer literacy, health literacy, traditional literacy, information literacy, science literacy, and media literacy. They also developed the eHealth Literacy Scale (eHEALS) to evaluate each individual’s perceptions toward eHealth literacy based on the Lily model [11].

The eHEALS has been shown to be reliable in diverse languages and has been validated in many countries [12-16]. Nevertheless, most of these evaluations were carried out in developed areas, and very few studies in developing areas were reported. eHEALS has also been translated into a traditional Chinese version in Taiwan [17]. For China, the largest developing country with a large rural population, rural-urban disparities exist in terms of cultural adoption, health care resource allocation, and personal health literacy [18,19]. Whether the eHEALS can be used to evaluate Chinese rural residents’ eHealth literacy is still unknown. We have two concerns about the existing traditional Chinese version of the eHEALS [17]; it cannot be reliable and valid in mainland China, especially for the Chinese rural population because of two reasons: (1) People living in the Chinese mainland were educated under a simplified Chinese environment. The long-term cultural divide between the Chinese mainland and Taiwan may produce certain semantic differences for specialized vocabularies. The language customs between Chinese mainland and Taiwan are obviously different. For instance, as a typical example of exotic vocabulary, the term “internet” was translated as “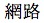 (wang lu)” in traditional Chinese in Taiwan. People who are not familiar with traditional Chinese in mainland China may deem “
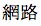
” as one type of physical infrastructure rather than the cyber platform. In the modern simplified Chinese context, the

“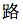 (lu)” of “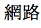 ” mostly refers to the physical road. The “internet” should be translated as “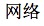 (wang luo),” which semantically emphasizes the network in simplified Chinese. (2) Even in the Chinese mainland, it is still necessary to testify whether the Chinese version of eHEALS is valid and reliable for the rural population. China has been facing an era of internal migration in the recent two decades [20-23]. With the inadequate development and limited work choices in rural areas, rural residents have to migrate to urban areas for better economic benefits, which, in turn, causes a significant issue of rural depopulation [23]. People who cannot migrate to urban areas mostly have a low literacy level and poor health status [24]. One report by the China Internet Network Information Center reveals that rural internet penetration was only 34.0% in mid-2017, while that in urban areas was 69.4% [19]. The perception and knowledge of ICT adoption among rural residents may lag behind those of urban residents. Given the discussion above, the eHEALS must be translated into simplified Chinese, and its psychometric analysis must be performed in the rural population.

The eHEALS was originally proposed to have a one-factor structure [11], which was supported by substantial evidence [12,16,17]. However, recent studies have found that it has a two-factor structure for Italian-speaking people [25], Israeli adults [26], and German adolescents [27]. Additionally, using confirmatory factor analysis, two studies have suggested that eHEALS may have a three-factor structure for baby boomers [28] and outpatients [29]. Although these studies used different statistical strategies, the inconsistent findings imply that the structure of the eHEALS may vary contextually. For instance, Hyde and colleagues indicated that the structure of eHEALS factors differs according to the task complexity [29]. This is true when testing the eHEALS among people living in metropolitan areas, since they have intrinsic modern knowledge distinguishable in terms of complexity levels in use of the eight items of eHEALS. However, the case might be vastly different among people with limited technological and medical literacy in developing areas. The one- or two-factor structure is rational in the latter scenario. Thus, to better understand Chinese rural people’s eHealth literacy status, the structure of the eHEALS for the Chinese rural population should be investigated in-depth.

Given the research question proposed above, this study aimed to develop a simplified Chinese version of the eHEALS (C-eHEALS) and evaluate its psychometric properties in a rural population. In this study, both the classical test theory and item response theory methods were adopted based on previous studies’ suggestions [25,30].

## Methods

### Procedure and Participants

In-person interviews on the theme of internet-mobile media usages and health outcomes in rural China were conducted in Chaotian, Sichuan Province, for three weeks in June 2017. Variables included in this study were one part of the entire questionnaire. As of 2017, Chaotian was a poverty-stricken county with 25 towns and a very low level of urbanization. The percentage of rural residents in Chaotian is more than 90% [31]. The quota sampling method was adopted, and each town was assigned 50 quotas considering its individual characteristics such as age, sex, education background, and residential districts. In total, 1250 questionnaires were delivered, and all interviews were conducted by trained local interviewers.

Before the survey, all participants received written information about the study and signed a consent form if they volunteered to participate in the study. When they completed the entire questionnaire, participants were given a small present as compensation. The questionnaires with major illogical, inaccurate, and missing answers accounting for more than 15% of total questions were identified as invalid. In total, 727 rural responses were valid in this survey. After the interviews, researchers randomly selected 30 participants for in-depth interviews about the health-related behaviors’ adoption and their influence on participants’ daily life. Of all valid responses, 543 participants who identified themselves as internet users were finally included in the analysis of the C-eHEALS.

### Measurement

#### Chinese Version of the eHealth Literacy Scale

This study focuses on the main measurement of the C-eHEALS (Multimedia Appendix 1). Like the original English version of the eHEALS, the C-eHEALS has eight items with response options on a five-point scale, ranging from 1 (strongly disagree) to 5 (strongly agree) [11]. The C-eHEALS was developed following the process of translation and adaptation of the instrument presented by the World Health Organization [32]. First, the eHEALS was translated into simplified Chinese by two bilingual researchers and then reviewed by a bilingual expert panel of four professionals in health communication studies and two rural medical professionals. After the expert panel evaluation, the translated instrument was revised and the complete C-eHEALS was generated. Thereafter, two independent native English translators with no knowledge of the eHEALS translated the C-eHEALS back to English. The resulting items were compared with the original items by the two English translators and the research team to identify possible semantic differences. In addition, the research team compared the simplified Chinese items with the traditional Chinese version of the eHEALS from the previous study [17] to confirm conceptual consistency.

#### Online Health Information–Seeking Behaviors

We used a multiple-choice question developed from the China Internet Network Information Center [19] to measure individuals’ online health information–seeking behaviors as the criterion measure of C-eHEALS. All participants were asked, “In the past 12 months, have you engaged in any of the following behaviors when you accessed the Internet?” We provided the following 11 choices: researched information about hospitals or doctors, researched information about physical exercise, researched information about smoking cessation, researched health or medical information, researched information about drinking cessation, read or shared health information via social media (eg, Weibo and WeChat), researched information about diet, wrote and shared health information via social media (eg, Weibo and WeChat), joined a specific disease internet community, purchased health care products online, and scheduled an appointment online. We added all answers to obtain one indicator—Scope of Online Health Information Seeking Behaviors (SOHISB)—to reflect the diversity in participants’ eHealth-related behaviors. Based on the number of behaviors selected, SOHISB ranged from 0 to 11.

#### Control Variables

For all analyses in this study, several demographic and socioeconomic variables (age, sex, and marital status) were controlled.

### Statistical Analysis

First, descriptive statistics, means, SDs, and percentages were calculated for the variables. Second, the C-eHEALS was evaluated according to the classical test theory approach. The reliability of the C-eHEALS was assessed using the Cronbach alpha coefficient (recommended value>.7) [33]. Subsequently, exploratory factor analysis and confirmatory factor analysis were conducted. In the exploratory factor analysis step, the Kaiser-Meyer-Olkin measure of sampling adequacy (recommended value>.6) and the Bartlett test of sphericity (should be statistically significant) [34] were used to test the factorability of C-eHEALS. Principal component analysis was then conducted to examine the latent properties of the eight observed items of C-eHEALS [35] and test whether the structure of the C-eHEALS has a unique pattern or is consistent with the Norman and Skinner one-factor structure [11]. Qualified factors via exploratory factor analysis should account for more than 40% of the total variance with eigenvalues>1 [36,37]. Moreover, a scree plot was used to determine the number of factors to be extracted. In the confirmatory factor analysis, we adopted structural equation modeling to evaluate the structure determined by exploratory factor analysis. The model’s goodness of fit was evaluated with the following: the Chi-square value to degrees of freedom ratio (χ^2^/df; recommended value<3) [38], comparative fit index (recommended value>.95), Tucker-Lewis index (recommended value >.95), root mean squared error of approximation (recommended value <.06), and standardized root mean squared residual (recommended value<.08) [39].

We thereafter tested other dimensions of the C-eHEALS’ psychometric properties using the item response theory approach. In this section, the graded response model [40], which is a generalization of the two-parameter logistic item response model (IRM) for ordinal data, was fit to the C-eHEALS. We chose IRM to help evaluate the C-eHEALS because IRM is more useful than classical test theory in providing information regarding item discriminability and difficulty [25]. Indeed, a recent study suggested that IRM should be used to evaluate the eHEALS properties [25]. Here, the graded response model was adopted over alternative IRMs because each item of the C-eHEALS has five ordered responses. In the graded response model, two types of parameters are generated: discrimination parameter alpha and difficulty or threshold parameter beta [40]. Although alpha indicates how strongly an item relates to a given latent trait theta, beta indicates the level of the latent variable a participant needs to endorse for the next higher response category, with a 50% probability. A larger beta suggests that a higher theta is required for participants to endorse a higher ordered response. Following Baker and Kim’s guidelines [41], alpha<.65 indicates low discriminability, .65-1.34 indicates moderate discriminability, and >1.34 indicates high discriminability. Next, item characteristic curves, which present the probability of participants at a given latent literacy level responding in a particular response category, were estimated for each item [42]. Then, item characteristic curves were transformed into item information curves to demonstrate how much information each item can provide. Thereafter, the test information function, which demonstrates the precision of the entire C-eHEALS along the latent trait continuum, was estimated by summing up all item information curves. In addition, the item characteristic curves were summed, in turn, to obtain the test characteristic curve, which represents the expected score of the C-eHEALS.

Subsequently, following the suggestion of a previous study [14], we also tested the criterion validity of the C-eHEALS via hypothesis testing. We hypothesized that the C-eHEALS score is positively associated with SOHISB among rural participants. Because SOHISB provides count data, Poisson regression was adopted to perform the estimation [43].

## Results

### Characteristics of the Rural Participants

Details of the characteristics of rural participants are listed in Table 1. Among the 543 participants, the mean age was 40.37 (SD 9.19) years, ranging from 18 to 70 years; men accounted for 58.56% of the participants. Most participants were married (83.43%). As for the highest education level, almost half of the participants listed junior middle school (46.49%), 27.81% listed primary school and below, 16.39% participants listed senior middle school, and 9.21% listed junior college and above. In terms of employment, approximately one-third of the participants (37.57%) had farming jobs, 17.31% had nonfarming jobs within the county, 27.44% had nonfarming jobs outside the county, 7.37% were unemployed, and 10.31% reported other jobs.

Although all samples were recruited in one county via quota sampling, which did not have an ideal representativeness, we compared the internet users in Chaotian and overall China as per a recent report [44]. At the end of 2015, 55.2% Chinese rural internet users were men [44], which was relatively equivalent to the sample in this study. However, the overall Chinese rural internet users showed a higher educational background (20.8% completed primary school and below, 51.9% completed junior middle school, 21.4% completed senior middle school, and 6% completed junior college and above) than the sample in Chaotian. Additionally, regarding the employment status, the overall Chinese rural internet users had fewer people in the farming occupation (15.8%) than the Chaotian sample. The differences between the Chaotian sample and overall Chinese internet users are not surprising as Chaotian, the survey site we chose, was a typical impoverished county when the investigation was conducted.

As for the 11 online health information–seeking behaviors, the least performed behavior was scheduling appointments online (3.31%) and the most was reading or sharing health information via social media (42.91%). As shown in Table 2, the mean of the items in the C-eHEALS ranged from 3.26-3.46 of 5.

**Table 1:** Characteristics of the rural participants (N=543).

Characteristics		n (%)
Age (years), mean (SD)		40.37 (9.19)
**Sex**		
	Male	318 (58.56)
	Female	225 (41.44)
**Marital status**		
	Married	453 (83.43)
	Not married	90 (16.57)
**Educational background**		
	Primary school and below	151 (27.81)
	Junior middle school	253 (46.59)
	Senior middle school	89 (16.39)
	Junior college and above	50 (9.21)
**Employment status**		
	Farming	204 (37.57)
	Working within the county	94 (17.31)
	Working outside the county	149 (27.44)
	Nonworking	40 (7.37)
	Other	56 (10.31)
**Online health information–seeking behavior**		
	Finding information about hospitals or doctors	96 (17.68)
	Finding information about physical exercises	159 (29.28)
	Finding information about smoking cessation	36 (6.63)
	Finding health or medical information	140 (25.78)
	Finding information about drinking cessation	30 (5.52)
	Reading or sharing health information via social media (eg, Weibo and Wechat)	233 (42.91)
	Finding information about diet	226 (41.62)
	Writing and sharing health information via social media (eg, Weibo and Wechat)	200 (36.83)
	Attending a specific disease Internet community	62 (11.42)
	Purchasing health care products online	38 (7.00)
	Online appointment	18 (3.31)

**Table 2:** Item means for the C-eHEALS in rural participants (N=543).

C-eHEALS items		Mean (SD)
C-eHEALS1	I know what health resources are available on the Internet	3.41 (0.76)
C-eHEALS2	I know where to find helpful health resources on the Internet.	3.32 (0.76)
C-eHEALS3	I know how to find helpful health resources on the Internet.	3.45 (0.73)
C-eHEALS4	I know how to use the Internet to answer my questions about health.	3.33 (0.79)
C-eHEALS5	I know how to use the health information I find on the Internet to help me.	3.46 (0.79)
C-eHEALS6	I have the skills I need to evaluate the health resources I find on the Internet.	3.34 (0.85)
C-eHEALS7	I can tell high quality health resources from low quality health resources on the Internet.	3.33 (0.78)
C-eHEALS8	I feel confident in using information from the Internet to make health decisions.	3.26 (0.87)

### Reliability and Exploratory Factor Analysis

**Figure 1:**
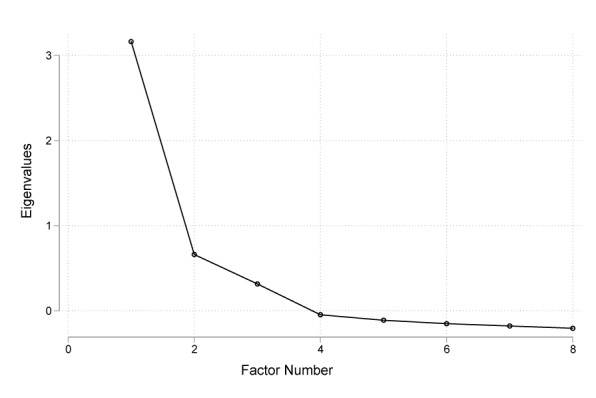
Scree plot for Chinese version of the eHealth Literacy Scale.

The C-eHEALS had excellent reliability (Cronbach alpha=.834). Both the Kaiser-Meyer-Olkin measure of sampling adequacy (.829; Table 3) and Bartlett test of sphericity (1556.34 (df=28), *P*<.001) showed a good fit to the data, allowing for exploratory factor analysis [34]. Exploratory factor analysis using principal component analysis resulted in a one-factor solution with an initial eigenvalue of 3.159, accounting for 91.8% of the variance (Table 3). The scree plot also showed a one-factor structure (Figure 1). As shown in Table 3, all items loaded above .5, varying from .576 (C-eHEALS1) to .706 (C-eHEALS3). Thus, a single factor was retained.

**Table 3:** Principal components analysis and Kaiser-Meyer-Olkin test of the C-eHEALS items.

C-eHEALS items	Factor loading	Kaiser-Meyer-Olkin value
C-eHEALS1	0.576	0.820
C-eHEALS2	0.629	0.814
C-eHEALS3	0.706	0.870
C-eHEALS4	0.635	0.820
C-eHEALS5	0.650	0.840
C-eHEALS6	0.582	0.839
C-eHEALS7	0.649	0.785
C-eHEALS8	0.590	0.842
Eigenvalue	3.159	N/A^a^
Cumulative explained variance, %	91.8	N/A
Overall Kaiser-Meyer-Olkin value	N/A	0.829

^a^N/A: not applicable.

### Confirmatory Factor Analysis

Confirmatory factor analysis was run to verify the one-factor structure obtained from exploratory factor analysis. Results suggested that the C-eHEALS has an excellent one-factor structure (χ^2^/df=1.813, comparative fit index=0.993, Tucker-Lewis index=0.985, root mean squared error of approximation=0.039, standardized root mean squared residual=0.022). Thus, the structure of the C-eHEALS is consistent with the original eHEALS proposed by Norman and Skinner [11] and the traditional Chinese version of the eHEALS [17].

### Item Response Theory: Graded Response Models

All eight items of the C-eHEALS were fit to a graded response model. The item parameter estimation and item fit statistics are displayed in Table 4. The discrimination parameters (alpha) ranged from 1.32 to 2.3, indicating that all items discriminated between low and high levels of eHealth literacy well. Only item C-eHEALS6 has a moderate alpha value (1.32), while the other seven items have high discriminability.

Difficulty parameter (beta) estimates indicated that the C-eHEALS is more sensitive at the lower range of latent trait theta because all mean beta (Beta*_M_*) values were lower than 0. The beta values of C-eHEALS1 and C-eHEALS5 were unevenly distributed across the trait range, indicating that most participants were unlikely to endorse lower response options. The other six items (C-eHEALS2, C-eHEALS3, C-eHEALS4, C-eHEALS6, C-eHEALS7, and C-eHEALS8) were distributed evenly across the trait range, suggesting that these items differentiate participants from low through high trait levels.

**Figure 2:**
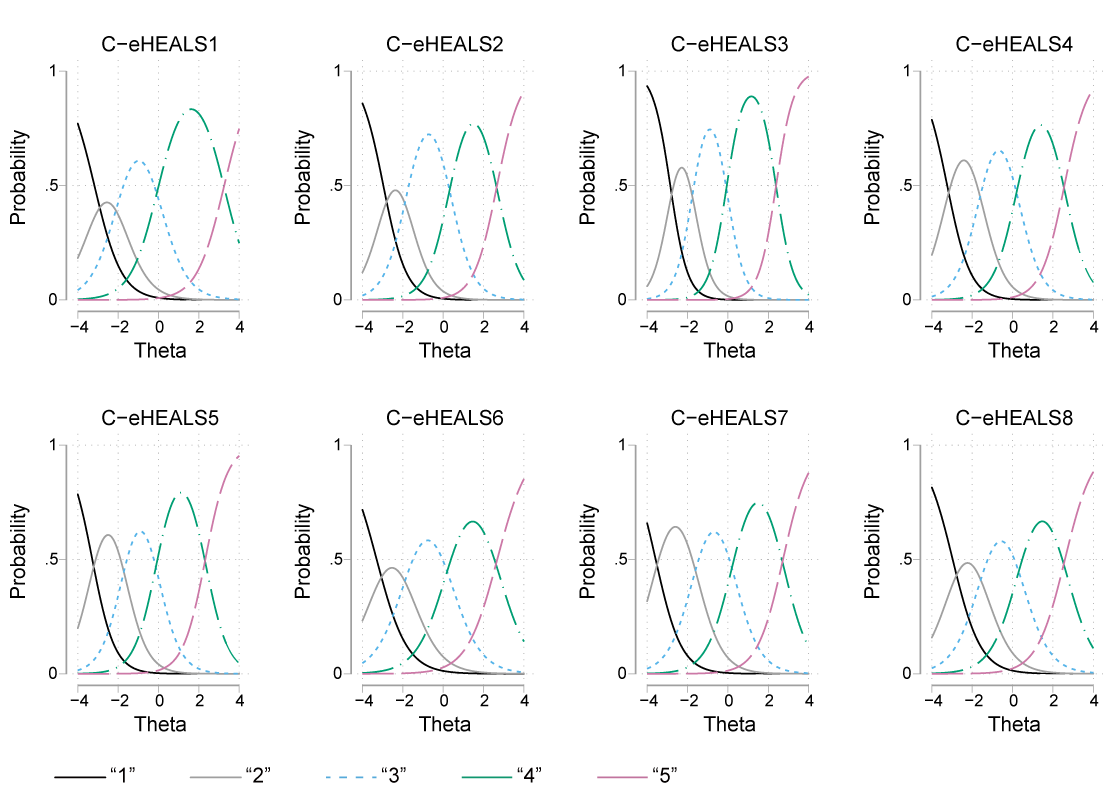
Item characteristic curves for each Chinese version of the eHealth Literacy Scale. Curves indicate the probability of participants at varying levels of eHealth literacy. C-eHEALS: Chinese version of the eHealth Literacy Scale.

In addition to these results, item characteristic curves are included in Figure 2. These plots show the probability that a participant selects a particular response category at a given level of the latent construct. It was observed that the response categories were distinguishable and monotonically related to the latent trait theta for all items.

**Figure 3:**
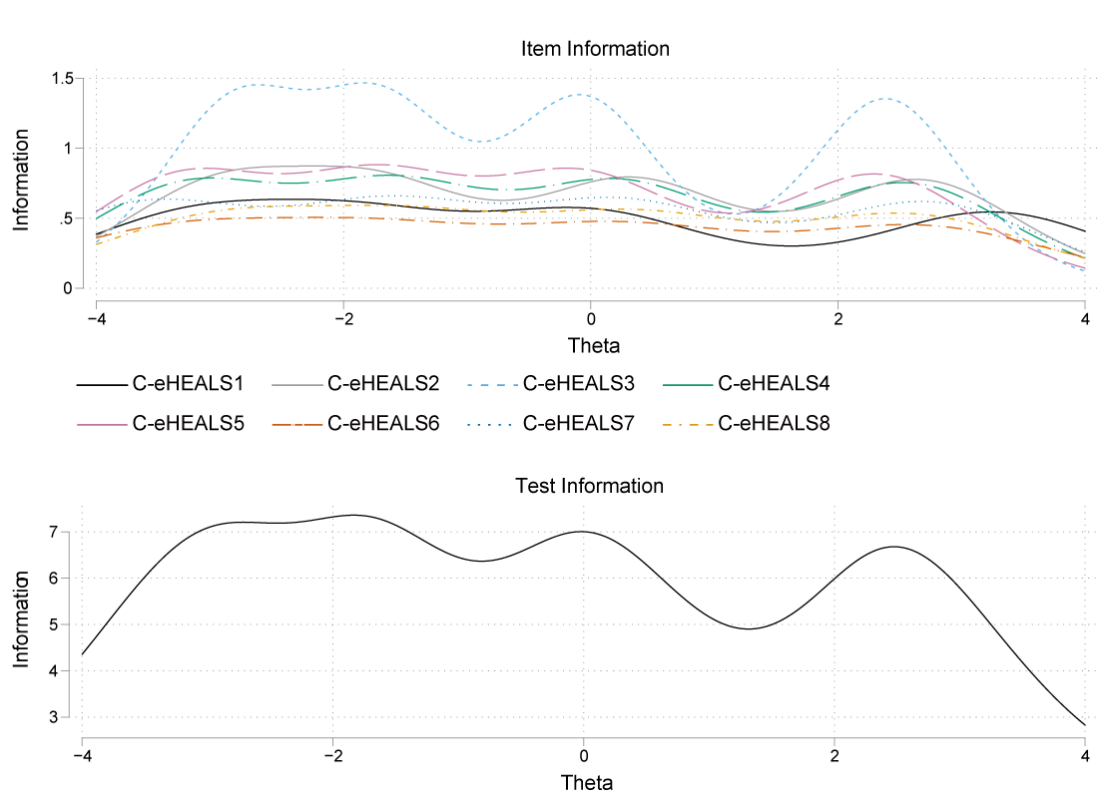
Item information curves and test information function for item characteristic curves. Curves indicate the amount of psychometric information (ie, the reciprocal of the standard error of measurement) provided by the instrument. C-eHEALS: Chinese version of the eHealth Literacy Scale.

**Figure 4:**
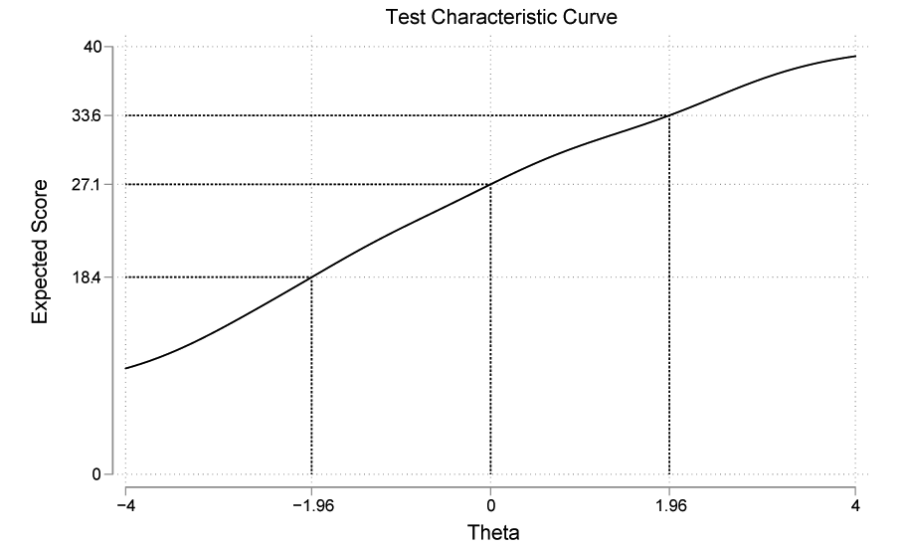
Test characteristic curve for Chinese version of the eHealth Literacy Scale.

Test information function, as reported in Figure 3, reveals that theta values<–3 and >2.5 are poorly represented relative to the rest of the trait range. This is true for seven items (C-eHEALS1, C-eHEALS2, C-eHEALS4, C-eHEALS5, C-eHEALS6, C-eHEALS7, and C-eHEALS8). Only the item information curve of C-eHEALS3 represented a significant fluctuation when theta levels are approximately between 1 and 3. Figure 4 presents the test characteristic curve, which indicates that 95% of randomly selected participants are expected to score between 18.4 and 33.6.

**Table 4:** Item Response Theory model parameters from C-eHEALS Graded Response Modelsa.

C-eHEALS items	Discrimination				Difficulty			
	alpha	SD	*P* value	beta_M_^b^	beta_1_	beta_2_	beta_3_	beta_4_
C-eHEALS1	1.46	0.15	<.001	–0.47	–3.18	–1.96	–0.01	3.26
C-eHEALS2	1.74	0.17	<.001	–0.43	–2.97	–1.77	0.33	2.67
C-eHEALS3	2.31	0.23	<.001	–0.53	–2.86	–1.72	0.06	2.39
C-eHEALS4	1.72	0.16	<.001	–0.51	–3.24	–1.59	0.23	2.57
C-eHEALS5	1.81	0.16	<.001	–0.69	–3.28	–1.70	–0.08	2.31
C-eHEALS6	1.32	0.13	<.001	–0.54	–3.30	–1.78	0.25	2.68
C-eHEALS7	1.55	0.15	<.001	–0.56	–3.58	–1.63	0.25	2.73
C-eHEALS8	1.43	0.14	<.001	–0.38	–2.97	–1.49	0.35	2.59

^a^Discrimination (alpha) refers to an item’s ability to discriminate between different latent levels of eHealth literacy (ie, theta). Difficulty parameters (beta) for responses on the 5-point Likert-type scale: 1 (from “strongly disagree” to “disagree”), 2 (from “disagree” to “neutral”), 3 (from “neutral” to “agree”), and 4 (from “agree” to “strongly agree”).

^b^Beta_M_: mean beta.

### Criterion Validity

The results of Poisson regression of the relationship between the C-eHEALS score and SOHISB are displayed in Tables 5 and 6. The odds ratio of the C-eHEALS score is significantly positive, which indicates that participants with higher C-eHEALS scores will have more diverse online health information–seeking behaviors. Hence, the C-eHEALS was shown to have good criterion validity for the Chinese rural population. In addition, coefficients of demographic variables (sex, age, and marital status) do not shown statistical significance, which means the SOHISB does not vary significantly with sex, age, and marital status. However, five dummy variables of socioeconomic status (educational background and employment status) presented significant positive associations with SOHISB. These results are consistent with the knowledge gap hypothesis [45] that implies that individuals will have more possibility to access the information channels to obtain useful information if they are living with higher socioeconomic status. More importantly, even after controlling for all these variables, the C-eHEALS score still plays a positive role in accessing internet technologies to seek health information. The findings in Tables 5 and 6 also suggest that eHealth literacy may have strong practical significance for its implementation to overcome social disparities.

**Table 5:** Poisson regression results of relationship between C-eHEALS score and SOHISB.

Item		Odds ratio	SD	T value	*P* value	95% CI
C-eHEALS score^a^		1.03	0.07	3.66	<.001	1.01-1.04
Female		1.08	0.07	1.20	.23	0.95-1.22
Age		0.99	0.00	–1.92	.054	0.98-1.00
Married		1.09	0.12	0.80	.42	0.88-1.34
**Educational Background (0=** **primary school and below)**						
	Junior middle school	1.29	0.10	3.21	.001	1.10-1.51
	Senior middle school	1.43	0.15	3.37	.001	1.16-1.76
	Junior college and above	1.41	0.17	2.79	.005	1.11-1.80
**Employment status (0=farming)**						
	Working within the county	1.48	0.13	4.64	<.001	1.26-1.75
	Working outside the county	1.25	0.10	2.86	.004	1.07-1.46
	Not working	1.18	0.15	1.27	.20	0.92-1.51
	Other	1.01	0.11	0.07	.95	0.81-1.25
Constant		1.00	0.27	–0.01	.99	0.59-1.70

^a^C-eHEALS: simplified Chinese version of the eHealth Literacy Scale.

**Table 6:** Poisson regression results.

Model Fit	Value
Observations, n	543
χ^2^ (df)	103.2 (11)
Log likelihood	–1062
PR^2^	0.0463

## Discussion

### Principal Findings and Implications

This study investigated the psychometric properties of the simplified C-eHEALS in a Chinese rural population via both classical test theory and item response theory approaches.

Classical test theory analyses demonstrated that the C-eHEALS has good reliability and validity for the rural population in China. The internal consistency of the C-eHEALS was .834, which was comparable to the original eHEALS’ alpha value of .88 reported by Norman and Skinner [11]. Exploratory factor analysis results revealed that the C-eHEALS has a one-factor structure, which is consistent with the structures of the original eHEALS [11] and its traditional Chinese version [17]. Surprisingly, the factor accounted for more than 91.8% of the variance, which is much higher than that reported in previous findings [11,14,16,17,25]. Furthermore, referring to previous studies’ suggestions [25,29], this one-factor structure also fit well in the confirmatory factor analysis.

Results of the item response theory revealed that response options could differentiate between latent trait levels of all eight C-eHEALS items. The entire instrument provides less information only at extremely low levels (theta<–3) and high levels (theta>2.5) of the latent trait. These results indicate that C-eHEALS is an excellent measure for capturing participants’ ability. Two items (C-eHEALS1 and C-eHEALS5) are more sensitive at the lower range of the latent trait, and the other six items represent excellent discriminability for participants from low through high trait levels.

This study also demonstrated that the C-eHEALS has good criterion validity. A previous study indicated that individuals with a higher level of health literacy will report a larger scope of health information sources [46]. Hence, the diversity of information access channels should be considered as one criterion of better literacy. We also hypothesized that the eHealth literacy score is positively associated with the SOHISB among rural populations. Indeed, controlling for confounding variables, Poisson regression results supported the hypothesis and revealed that rural people’s information-seeking behavior could be cultivated with adequate eHealth literacy.

The eHEALS is a validated instrument in diverse language environments [14,16,17,25,27]. In mainland China, it was first introduced in 2013 [47] and has received attention in recent years [48,49]. However, those studies were limited to reporting sophisticated psychometric properties of the eHEALS [47-49]. In addition, rural populations were ignored in previous research. Given the currently targeted poverty alleviation strategies, the campaign launched by the Chinese central government to enhance residents’ health literacy status over the next decade [8], and the obvious internet access gap between rural and urban areas [19], both community- and county-level health promotion campaigns should emphasize on health education for rural populations to mitigate large rural-urban disparities.

Health information diffused via the internet should be appropriately evaluated by individual internet users, which can be strengthened by health literacy. For future health literacy-related studies concerning Chinese rural populations, this study provides a useful instrument that can be adopted in survey studies. Moreover, highlighting aspects of health literacy specific to the internet context (eg, practical skills) over other aspects such as the perception description in the eHEALS should be considered for future research.

Findings about the criterion validity also revealed that eHealth literacy is a key element to promote rural residents’ access to ICTs for health-related information. Enhancing eHealth literacy might help rural residents overcome their inadequate resource acquisition capacities restricted by local economic recession. It is worth noting that eHealth literacy may also lead to a new digital divide between rural and urban populations. Besides the information-seeking behavior, some other online health-related practices, like health management [4] and mobile app-assisted self-care [50], may present varied implementation practices among people with different socioeconomic statuses. Future studies should design comparisons between populations living in areas with different levels of urbanization.

### Limitations

There are two main limitations in this study. First, all participants were recruited from one poverty-stricken county in China via quota sampling, which cannot well represent the diverse situations of China’s rural-urban disparities and the entire Chinese rural population. The psychometric properties of the C-eHEALS may vary under different economic development statuses. Hence, future studies aiming to replicate our findings in other samples are highly encouraged. Second, this study had a cross-sectional design, and hence, we were unable to calculate test-retest reliability or predictive validity estimates [51]. Future studies may address this limitation via longitudinal designs.

### Conclusions

The C-eHEALS was found to have a robust one-factor structure with excellent discriminability among the Chinese rural population. This scale is helpful for health education practitioners and health professionals to properly measure and understand rural people’s eHealth literacy before launching health campaigns. We hope to encourage health researchers who conduct studies in eHealth to carefully investigate policy effects on rural people.

## Appendix

Multimedia Appendix 1 The simplified Chinese version of eHealth Literacy Scale (eHEALS).

## Abbreviations

C-eHEALS: simplified Chinese version of the eHealth Literacy Scale
eHEALS: eHealth Literacy Scale
eHealth: electronic health
ICTs: information communication technologies
IRM: item response model
SOHISB: Scope of Online Health Information Seeking Behaviors
