# Supplementary material for: The Psychometric Properties of the Chinese eHealth Literacy Scale (C-eHEALS) in a Chinese Rural Population: Cross-Sectional Validation Study
Source: J Med Internet Res. 2019 Oct 22;21(10):e15720. doi: 10.2196/15720 (PMC6914234; doi:10.2196/15720)
Supplement: Multimedia Appendix 1 [file jmir_v21i10e15720_app1.pdf]

Multimedia Appendix 1: The simplified Chinese version of *eHealth Literacy Scale* (eHEALS)

对于以下陈述，请选择最符合您情况的数字（请逐一评价）

	非常不同意	不同意	不确定	同意	非常同意
C-eHEALS1:我知道网上可以找到什么样的健康资源….....	1□	2□	3□	4□	5□
C-eHEALS2:我知道网上去哪可以找到有用的健康资源....	1□	2□	3□	4□	5□
C-eHEALS3:我知道如何在网上找到有用的健康资源........	1□	2□	3□	4□	5□
C-eHEALS4:我知道怎样利用网络来解答自己的健康问题.	1□	2□	3□	4□	5□
C-eHEALS5:我知道如何利用网上的健康信息来帮助自己.	1□	2□	3□	4□	5□
C-eHEALS6:我具备评价网上健康资源好坏的能力...........	1□	2□	3□	4□	5□
C-eHEALS7:我能区分高质量与低质量的网上健康资源.....	1□	2□	3□	4□	5□
C-eHEALS8:我有使用网络信息来做出健康决策的自信.....	1□	2□	3□	4□	5□
